# Association between changes in the lung-to-head ratio and postnatal survival in fetuses with congenital diaphragmatic hernia

**DOI:** 10.1016/j.clinsp.2026.101073

**Published:** 2026-09-07

**Authors:** Yiqing Yu, Qingwei Qi, Xiya Zhou, Na Hao, Qing Dai, Hua Meng, Yunshu Ouyang, Yixiu Zhang, Hui You, Zhenghong Li, Shan Jian, Lishuang Ma, Ying Wang, Lijian Pei, Yan Lü, Yulin Jiang

**Affiliations:** a Department of Obstetrics and Gynecology, National Clinical Research Center for Women’s Health and Obstetric and Gynecologic Diseases, Peking Union Medical College Hospital, Chinese Academy of Medical Sciences & Peking Union Medical College, Beijing, China; b Department of Ultrasound, Peking Union Medical College Hospital, Chinese Academy of Medical Sciences & Peking Union Medical College, Beijing, China; c Department of Radiology, Peking Union Medical College Hospital, Chinese Academy of Medical Sciences & Peking Union Medical College, Beijing, China; d Department of Pediatrics, Peking Union Medical College Hospital, Chinese Academy of Medical Sciences & Peking Union Medical College, Beijing, China; e Department of Neonatal Surgery, Children’s Hospital of Capital Institute of Pediatrics, Capital Institute of Pediatrics, Beijing, China; f Department of Anesthesiology, Peking Union Medical College Hospital, Chinese Academy of Medical Sciences & Peking Union Medical College, Beijing, China

**Keywords:** Congenital diaphragmatic hernia, Observed-to-expected lung-to-head ratio, Longitudinal change, Fetus, Prognosis

## Abstract

**Introduction:**

To evaluate the association between longitudinal changes in Observed-to-Expected Lung-to-Head Ratio (O/E LHR) measured by ultrasound and postnatal survival in fetuses with Congenital Diaphragmatic Hernia (CDH).

**Methods:**

A total of 74 pregnant women carrying fetuses with CDH were retrospectively analyzed. The change in O/E LHR (ΔO/E LHR) was assessed as a continuous variable using univariate and multivariate logistic regression analyses. Hernia lateralization and liver herniation were combined into a single categorical variable to account for their clinical and statistical correlation. Receiver Operating Characteristic (ROC) analysis was performed to evaluate the discriminative ability of ΔO/E LHR for survival.

**Results:**

The majority of patients (86% and 74% according to the first and last ultrasound examinations, respectively) in this cohort had mild CDH, with an overall survival rate of 73.0%. ΔO/E LHR differed significantly between survivors and non-survivors (13.32±22.09 vs. -13.93±17.59, *p* < 0.001). In multivariate analysis, ΔO/E LHR (OR = 1.089, 95% CI 1.024–1.158; *p* = 0.007), gestational age at diagnosis (OR = 1.462, 95% CI 1.041–2.053; *p* = 0.029), and the combined variable of hernia lateralization and liver herniation (*p* = 0.011) were associated with survival. ROC analysis of ΔO/E LHR yielded an AUC of 0.836 (95% CI 0.725–0.948).

**Conclusion:**

In this single-center cohort of fetuses with predominantly mild CDH, ΔO/E LHR was associated with postnatal survival and showed good discriminative performance. Gestational age at diagnosis and the combined variable of hernia lateralization and liver herniation were also associated with outcomes. However, estimates for individual anatomical subgroups were imprecise because of the limited number of cases and should not be used for individual risk prediction. These findings suggest that longitudinal assessment of O/E LHR may be more informative than a single measurement in this specific population. Further validation in larger cohorts is warranted.

## Highlights

• Gestational change in observed-to-expected lung-to-head ratio is associated with survival in fetuses with mild CDH.
• Positive change in observed-to-expected lung-to-head ratio indicates improved prognosis.
• Longitudinal assessment of observed-to-expected lung-to-head ratio provides additional prognostic value.

## Introduction

Congenital Diaphragmatic Hernia (CDH) is a developmental defect of the diaphragm resulting in herniation of the abdominal organs into the thoracic cavity. CDH is one of the most common congenital defects, with an estimated incidence of 1 in 2500–5000 births.^1^, ^2^, ^3^ The compression caused by CDH, combined with other intrinsic factors, leads to pulmonary hypoplasia, resulting in a mortality rate as high as 80%.^4^^,^^5^ However, the overall prognosis has improved significantly in recent decades owing to advances in prenatal evaluation and appropriate treatments.^6^, ^7^, ^8^, ^9^, ^10^

The Observed-to-Expected Lung-to-Head Ratio (O/E LHR) measured by Ultrasound (US) is widely used in clinical practice to estimate fetal lung size and assess disease severity.^11^ The LHR is calculated by dividing the lung area contralateral to the hernia by the fetal head circumference. An O/E LHR < 25% in left-sided CDH or an O/E LHR < 45% in right-sided CDH indicates a poor outcome.^6^ However, this measurement reflects only the contralateral lung and may not fully capture the overall severity of pulmonary hypoplasia.^12^^,^^13^

Nevertheless, precise prenatal prognostication remains a clinical challenge. Although the longitudinal trajectory of O/E LHR has been recognized, its prognostic significance is currently obscured by inconsistent evidence in the literature.^14^, ^15^, ^16^ Furthermore, there is a critical need to clarify whether the dynamic evolution of lung growth provides additional predictive insight beyond traditional static assessments. Consequently, the present study aims to evaluate the potential clinical relevance of gestational changes in O/E LHR (∆O/E LHR) and their specific association with survival to discharge in a cohort of fetuses with CDH.

## Materials and methods

### Setting and patients

This retrospective study included a total of 78 pregnant women carrying fetuses with CDH from January 2019 to April 2024. Detailed ultrasound examination of the fetal lungs was performed at the first clinical visit upon referral of each pregnant woman to our hospital, which is a tertiary medical institution and a center for maternal-fetal medicine. Amniocentesis for chromosomal karyotyping and chromosomal microarray analysis was performed if not completed previously. Monthly follow-up US was performed until delivery. After birth, the newborns were immediately intubated for mechanical ventilation and transferred to the neonatal intensive care unit. Surgery was performed after evaluation. The post-birth intervention protocol has been published previously.^10^ Fetuses with chromosomal abnormalities (n = 1), those lost to follow-up (n = 2), and pregnancies terminated on maternal request (n = 1) were excluded. A total of 74 fetuses with CDH were ultimately included in the study. This study was approved by the institutional review board (IRB No. I-24PJ1052) and followed the STROBE Statement.

### Data collection and outcome measures

Clinical characteristics ‒ including baseline, prenatal, and perinatal details ‒ were retrospectively retrieved from medical records. Baseline characteristics included demographic features, medical history, and pregnancy-related complications. Prenatal characteristics included gestational age at the time of CDH diagnosis and US findings. US examinations were performed by four experienced sonographers who had expertise in prenatal diagnosis. The longest axis method was used to calculate the O/E LHR.^17^^,^^18^ For descriptive purposes, CDH severity was categorized based on O/E LHR. Mild left CDH was defined as an O/E LHR ≥ 35%, and mild right CDH was defined as an O/E LHR ≥ 45%.^18^ Cases with O/E LHR below these thresholds were classified as moderate to severe CDH. The change in O/E LHR (ΔO/E LHR) was calculated as: ΔO/E LHR = O/E LHR at the last ultrasound examination ‒ O/E LHR at the first ultrasound examination, and was expressed in percentage points. For example, if the O/E LHR increased from 40% at the first examination to 50% at the last examination, the ΔO/E LHR was +10 percentage points. Additional variables including the right or left lateralization of the hernia, the position of the liver, and the presence of other US-detected anomalies were recorded. The perinatal characteristics recorded in this study included gestational age at delivery and neonatal sex. The primary outcome was survival, defined as survival to discharge without the need for respiratory support following surgical repair.

### Data analysis

Categorical variables are expressed as frequencies and percentages. Continuous variables are expressed as the means with Standard Deviations (SDs) or medians with Interquartile Ranges (IQRs), as appropriate, based on their distribution after the Shapiro-Wilk test. Univariate logistic regression analysis was performed to evaluate the association between each clinical variable and survival. Variables considered clinically relevant or supported by prior evidence were included in the multivariate analysis. Multivariate analysis was conducted using a binary logistic regression model with all selected variables entered simultaneously (the enter method) to reduce the risk of model overfitting and to ensure the inclusion of clinically important confounders. Given the strong clinical and statistical correlation between hernia lateralization and liver herniation, these variables were combined into a single categorical variable with three clinically relevant groups: left-sided CDH without liver herniation, left-sided CDH with liver herniation, and right-sided CDH with liver herniation. This approach was used to minimize multicollinearity and to better reflect clinical characteristics. ΔO/E LHR was analyzed as a continuous variable in all regression models. To ensure consistency, only cases with complete longitudinal data were included in the multivariate analysis. Receiver Operating Characteristic (ROC) analysis was performed to evaluate the discriminative ability of ΔO/E LHR for survival. The Area Under the Curve (AUC) was calculated with corresponding 95% Confidence Intervals. All statistical tests were two-sided, and a *p*-value < 0.05 was considered statistically significant. Statistical analyses were performed using SPSS version 29.0 (IBM Corp., Armonk, NY, USA).

## Results

### Patients

The majority of fetuses in this cohort were classified as having mild CDH, accounting for 86% and 74% at the first and last ultrasound examinations, respectively. The overall survival rate was 73.0% (54/74). No significant differences in baseline characteristics were observed between the survivor and non-survivor groups (Table 1).

**Table 1:** Baseline maternal and gestational characteristics of fetuses with CDH in the survivor and non-survivor groups.

Clinical characteristics	Survivors (n = 54)	Non-survivors (n = 20)	*p*-value
Maternal age at delivery, years, mean ± SD	31.61 ± 4.12	31.75 ± 5.10	0.903
Nullipara, n (%)	36 (66.67)	10 (50.00)	0.193
History of recurrent CDH in previous pregnancies, n (%)	0 (0.00)	2 (10.00)	>0.999
Diabetes mellitus, n (%)	11 (20.37)	6 (30.00)	0.385
Hypertension, n (%)	5 (9.26)	0 (0.00)	>0.999
Other pregnancy complications, n (%)^a^	4 (7.41)	2 (10.00)	0.718

a Other pregnancy complications included lymphoma (n = 1), systemic lupus erythematosus (n = 1), undifferentiated connective tissue disease (n = 1), thrombophilia (n = 1), and dichorionic diamniotic twins (n = 2). CDH, Congenital Diaphragmatic Hernia.

### Association of clinical variables with survival

Univariate logistic regression analysis (Table 2) identified several variables associated with survival: 1) gestational age at CDH diagnosis; 2) mild CDH classification at the last examination; 3) ΔO/E LHR; 4) the combined variable of hernia lateralization and liver herniation; and 5) concurrent ultrasound abnormalities (detailed in Table S1). In the multivariate logistic regression analysis, gestational age at diagnosis (OR = 1.462, 95% CI 1.041–2.053; *p* = 0.029), ΔO/E LHR (OR = 1.089, 95% CI 1.024–1.158; *p* = 0.007), and the combined variable of hernia lateralization and liver herniation (overall *p* = 0.011) remained associated with survival. Specifically, compared with left-sided CDH without liver herniation, right-sided CDH with liver herniation was associated with reduced survival (OR = 0.017, 95% CI 0.001–0.275; *p* = 0.004).

**Table 2:** Associations between prenatal and perinatal characteristics with survival in fetuses with CDH.

Clinical characteristics	Survivors (n = 54)	Non-survivors (n = 20)	Univariate analysis		Multivariate analysis	
			*p*-value	OR (95% CI)	*p*-value	OR (95% CI)
Prenatal characteristics						
Gestational age at CDH diagnosis, weeks, median (IQR)	24 [22, 29]	23 [22, 24]	**0.027**	**1.211 (1.022, 1.435)**	**0.029**	**1.462 (1.041, 2.053)**
US evaluation						
Gestational age at the first examination, weeks, median (IQR)^a^	29 [26, 32]	29 [27, 32]	0.891	1.009 (0.886, 1.105)		
Mild CDH according to the first examination, n (%)^a^	48 (90.57)	16 (84.21)	0.454	1.800 (0.386, 8.389)		
Gestational age at the last examination, weeks, median (IQR)^b^	36 [35, 36]	36 [34, 36]	0.284	1.141 (0.896, 1.454)		
Mild CDH according to the last examination, n (%)^b^	46 (92.00)	9 (56.25)	**0.003**	**8.944 (2.160, 37.044)**	0.532	2.066 (0.213, 20.079)
Interval duration between examinations, weeks, median (IQR)^b^	6.0 [3.0, 9.0]	6.5 [5.3, 7.8]	0.797	0.980 (0.837, 1.146)		
ΔO/E LHR, %, mean ± SD^b^	13.32 ± 22.09	−13.93 ± 17.59	**<0.001**	**1.075 (1.032, 1.120)**	**0.007**	**1.089 (1.024, 1.158)**
Hernia lateralization and liver herniation (combined), n (%)						
Left CDH without liver herniation (reference), n (%)	45 (83.33)	8 (40.00)	**0.002**		**0.011**	
Left CDH with liver herniation, n (%)	1 (1.85)	4 (20.00)	**0.008**	**0.044 (0.004, 0.451)**	0.105	0.085 (0.004, 1.682)
Right CDH with liver herniation, n (%)	8 (14.81)	8 (40.00)	**0.006**	**0.178 (0.052, 0.611)**	**0.004**	**0.017 (0.001, 0.275)**
Concurrent US abnormalities, n (%)	2 (3.70)	6 (30.00)	**0.006**	**0.090 (0.016, 0.494)**	0.314	0.153 (0.004, 5.913)
Perinatal characteristics						
Gestational age at delivery, weeks, median (IQR)	37 [37, 38]	37 [37, 38]	0.629	0.918 (0.650, 1.297)		
Preterm labor, n (%)	9 (16.67)	3 (15.00)	0.863	1.133 (0.274, 4.692)		
Male neonate, n (%)	29 (53.70%)	12 (60.00%)	0.629	0.773 (0.273, 2.193)		

A *p*-value < 0.05 is considered significant, and significant values are marked with bold text.

a Data available for 53 survivors and 19 non-survivors.

b Data available for 50 survivors and 16 non-survivors. Interval duration between examinations was defined as the gestational age interval between the first and last O/E LHR assessments. CDH, Congenital Diaphragmatic Hernia; CI, Confidence Interval; IQR, Interquartile Range; ΔO/E LHR, Change in Observed-to-Expected Lung-to-Head Ratio; OR, Odds Ratio; US, Ultrasound.

### Longitudinal changes in O/E LHR

**Fig. 1:**
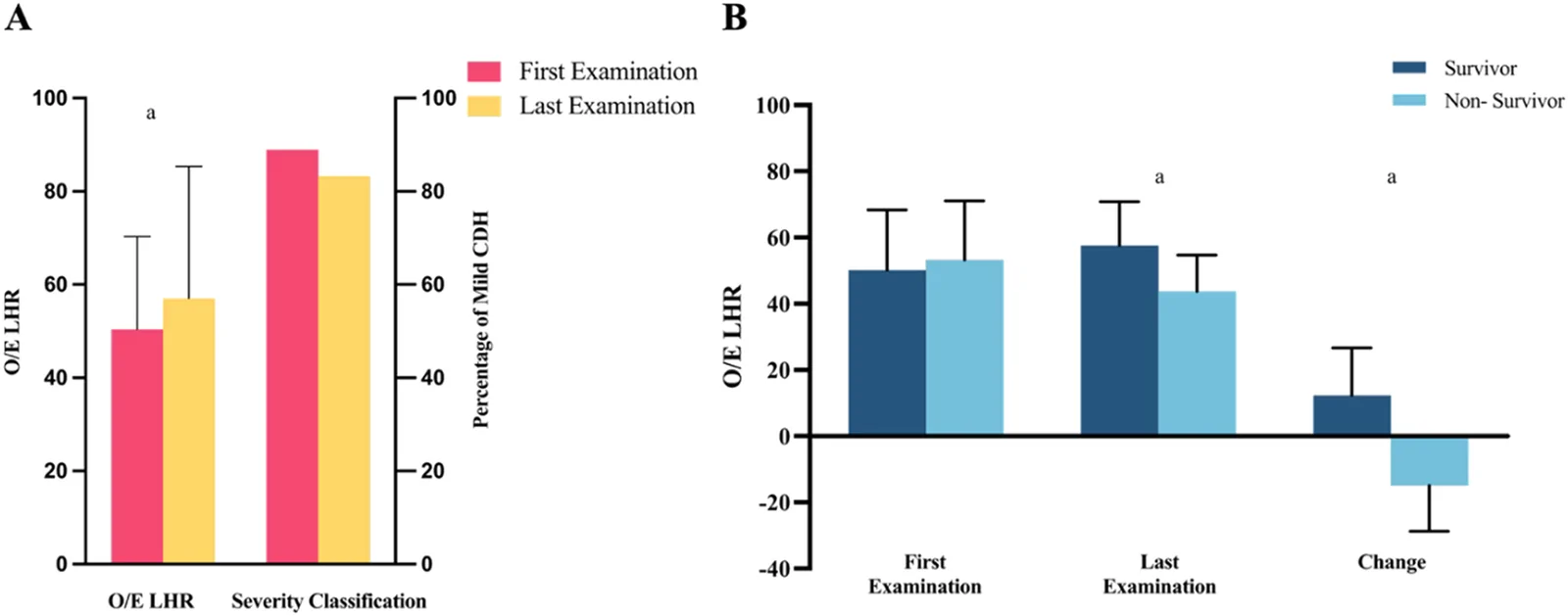
Longitudinal changes in O/E LHR during pregnancy. (A) Comparison of O/E LHR values and severity classifications between the first and last ultrasound examinations in the entire cohort. (B) Comparison of first O/E LHR, last O/E LHR, and ΔO/E LHR between survivors and non-survivors. ^a^ Statistically significant.

The O/E LHR measurements were recorded longitudinally during pregnancy. A small but statistically significant difference was observed between these two timepoints in the overall cohort (50.41 [42.70, 70.30] vs. 57.00 [41.18, 85.35], *p* = 0.049), whereas the distribution of severity categories remained similar (mild CDH: 88.9% vs. 83.3%, *p* = 0.206) (Fig. 1A). No significant difference in O/E LHR at the first examination was observed between survivors and non-survivors (50.22 [43.40, 68.30] vs. 53.20 [41.80, 71.00], *p* = 0.838). In contrast, O/E LHR at the last examination was higher in survivors than in non-survivors (64.10 [47.40, 89.00] vs. 43.70 [27.70, 54.70], *p* = 0.013) (Fig. 1B). The mean ΔO/E LHR differed between the two groups, with a positive change observed in the survivor group (13.32 ± 22.09) and a negative change in the non-survivor group (−13.93 ± 17.59; *p* < 0.001) (Fig. 1B). Receiver operating characteristic analysis of ΔO/E LHR yielded an area under the curve of 0.836 (95% CI 0.725–0.948).

## Discussion

This single-center study evaluated the prognostic value of lung growth trajectories in a cohort of CDH fetuses characterized by a predominance of mild cases. Our findings demonstrated that ΔO/E LHR, gestational age at diagnosis, and the combined variable of hernia lateralization and liver herniation are independently associated with postnatal survival. These findings should be interpreted within the context of a relatively low-risk cohort, in which the ability of a single O/E LHR measurement to discriminate outcomes may be limited.

### Longitudinal changes in O/E LHR and their association with survival

Although the association between a single O/E LHR value and the prognosis of CDH has been well established, relatively few studies have examined the longitudinal change in O/E LHR during pregnancy, and the findings of those few studies have been inconsistent.^14^, ^15^, ^16^ Previous studies have suggested that O/E LHR values at diagnosis and at later gestational ages show comparable predictive performance, supporting its role in early risk stratification.^19^, ^20^, ^21^

In the present cohort, O/E LHR values and severity classification remained broadly similar between the first and last examinations, which may reflect the relatively homogeneous distribution of predominantly mild CDH cases. Nevertheless, a difference in ΔO/E LHR between survivors and non-survivors was observed. A similar pattern was reported by Antolin et al.,^16^ who found that the O/E LHR of non-survivors trended downward over time, whereas survivors showed relatively stable measurements.

Receiver operating characteristic analysis of ΔO/E LHR in our cohort yielded an AUC of 0.836. A threshold of 0 was close to the Youden index and provided a relatively balanced sensitivity and specificity profile in this dataset (approximately 74%–78% sensitivity and 81.2% specificity). Given its simplicity and intuitive interpretation (increase versus decrease in ΔO/E LHR), this threshold may serve as a candidate cutoff for future investigation. However, because both the threshold and the corresponding performance metrics were derived and evaluated within the same cohort, they should be considered exploratory and hypothesis-generating. External validation in larger and more diverse cohorts is required before any clinical application.

### Gestational age at CDH diagnosis and survival

Regarding gestational age, some studies have suggested that an earlier diagnosis, particularly before 25-weeks of gestation, is associated with increased mortality, whereas others have reported no significant association with postnatal outcomes.^17^^,^^22^, ^23^, ^24^, ^25^, ^26^, ^27^, ^28^, ^29^, ^30^, ^31^ However, these findings should be interpreted with caution, as many of these studies were limited by small sample sizes, and gestational age has often been analyzed as a categorical rather than a continuous variable, which may reduce statistical power.

In the present study, gestational age at diagnosis was analyzed as a continuous variable, and an association with survival was observed after adjustment for other clinical factors. This finding is in line with previous reports suggesting that earlier diagnosis may be linked to more severe disease.^26^^,^^27^ One possible explanation is that earlier-diagnosed cases may represent fetuses with larger diaphragmatic defects and more pronounced impairment of pulmonary development, including both lung growth and vascularization, which are known contributors to pulmonary hypoplasia and persistent pulmonary hypertension.^26^^,^^27^ Nevertheless, this interpretation should be approached cautiously, as gestational age at diagnosis may also be influenced by ultrasound detection practices and capabilities.

### Hernia lateralization and liver herniation

Hernia lateralization and liver herniation are well-recognized clinical factors associated with disease severity and outcomes in patients with CDH. In the present study, these two variables were strongly correlated and are closely linked from a clinical perspective. Therefore, they were combined into a single categorical variable in the multivariate analysis to better capture their joint clinical relevance while reducing potential collinearity.

Under this combined variable, fetuses with left CDH without liver herniation had the most favorable outcomes, whereas those with left CDH with liver herniation and right CDH with liver herniation showed poorer survival. This pattern is consistent with the findings of previous studies, in which liver herniation was associated with reduced survival in fetuses with CDH.^32^^,^^33^ In addition, liver herniation has been linked to increased disease severity and adverse outcomes even after accounting for lung size, supporting its role as an independent marker of prognosis.^34^ Right-sided CDH is also frequently associated with increased liver herniation and exacerbated impairment of lung development, which may further contribute to poorer outcomes.^35^

However, the distribution across categories was uneven, with limited numbers in some subgroups. As reflected by the wide confidence intervals, these estimates should be interpreted cautiously, and the findings primarily support the direction of association rather than providing precise risk estimates. Subgroup analyses were not performed because of the small sample sizes; instead, the analysis was restricted to the overall cohort to ensure more robust and interpretable results.

### Limitations

Our study had several limitations. First, this was a single-center retrospective study with a relatively small sample size, which may limit the generalizability of the findings. In particular, the cohort was predominantly composed of fetuses with mild CDH; therefore, the results should be interpreted within this specific clinical context. Second, the uneven distribution of cases across subgroups may have resulted in unstable estimates, as reflected by the wide confidence intervals observed in some analyses. Third, although multivariate analysis was performed to adjust for potential confounders, residual confounding cannot be excluded. Taken together, these limitations indicate that the present findings should be considered exploratory and hypothesis-generating results, and further validation in larger and multicenter cohorts is warranted.

## Conclusions

In conclusion, in this single-center cohort of fetuses with predominantly mild CDH, ΔO/E LHR was associated with postnatal survival and showed better discriminatory performance than a single O/E LHR measurement did. Gestational age at diagnosis and the combined variable of hernia lateralization and liver herniation were also associated with survival after adjustment for potential confounders. However, estimates for individual anatomical subgroups were imprecise because of the limited number of cases and should not be used for individual risk prediction. These findings suggest that longitudinal assessment of O/E LHR may provide additional information beyond a single measurement in this specific population. Further validation in larger, multicenter, and more heterogeneous cohorts is needed to confirm these findings.

## Authors’ contributions

Yiqing Yu: Data curation, formal analysis, writing-original draft. Qingwei Qi, Xiya Zhou, Na Hao, Qing Dai, Hua Meng, Yunshu Ouyang, Yixiu Zhang, Hui You, Zhenghong Li, Shan Jian, Lishuang Ma, Ying Wang, Lijian Pei: Data curation. Yan Lü: Conceptualization, funding acquisition, methodology, writing-review & editing. Yulin Jiang: Conceptualization, data curation, writing-review & editing. All the authors have read and approved the manuscript.

## Informed consent

Written informed consent was obtained from all participants.

## Statement of ethics

This study adheres to the principles of the Declaration of Helsinki. The study was approved by the Institutional Review Board of PUMCH (IRB No. I-24PJ1052).

## Declaration of generative AI and AI-assisted technologies in the writing process

The authors did not use generative AI or AI-assisted technologies in the writing process of this manuscript.

## Funding

This work was supported by the Peking Union Medical College Hospital Outstanding Young Talent Development Program (Category C) [UBJ10395], the National High-Level Hospital Clinical Research Funding [2022-PUMCH-A-088 and 2022-PUMCH-B-076] and the National Key Clinical Specialty Construction Project [U114000].

## Data availability statement

In keeping with hospital and IRB policies, the datasets generated and/or analyzed during the current study are not publicly available. However, they are available from the corresponding author upon reasonable request.

## Conflicts of interest

The authors declare no conflicts of interest.

## Appendix Supplementary materials

Image, application 1
