# Supplementary material for: Association between changes in the lung-to-head ratio and postnatal survival in fetuses with congenital diaphragmatic hernia
Source: Clinics (Sao Paulo). 2026 Sep 7;81:101073. doi: 10.1016/j.clinsp.2026.101073 (PMC13572760; doi:10.1016/j.clinsp.2026.101073)
Supplement: Supplementary file 1 [file mmc1.docx]

**CLINICS-D-25-00660**

**Supplementary information**

**Table S1** Ultrasound abnormalities concurrent with fetal CDH.

| **Case No.** | **Concurrent ultrasound abnormalities** |
| --- | --- |
| 20 | Cleft lip, ventricular septal defect |
| 28 | Left pelvic kidney, postaxial polydactyly of the right hand, bilateral microtia |
| 39 | Bilateral adducted thumb |
| 53 | Cervical vertebral dysplasia, abnormal umbilical-portal-inferior cava vein connection |
| 59 | Ventricular septal defect |
| 60 | Thymus mass |
| 68 | Massive tricuspid regurgitation |
| 76 | Massive pleural effusion |
